# The association of household conditions with the development of active pulmonary TB among in households with a previous TB patient: a community-based case–control study

**DOI:** 10.1186/s12890-026-04434-5

**Published:** 2026-06-26

**Authors:** Ringga Rahmi Prima, Alan F. Geater, Defriman Djafri, Virasakdi Chongsuvivatwong

**Affiliations:** 1 Department of Epidemiology, Faculty of Medicine, Prince of Songkla University, Hatyai, Songkhla, , Thailand; 2 Department of Epidemiology and Biostatistics, Faculty of Public Health, Andalas University, Padang, Indonesia

**Keywords:** Previous household TB patient, Pulmonary tuberculosis, Household conditions, Construction materials

## Abstract

**Background:**

Poor housing has been linked to tuberculosis (TB), yet details of living conditions are less well studied.

**Methods:**

This community-based case–control study examined household and individual factors associated with active pulmonary TB among households with a prior TB patient in Padang City, West Sumatra, Indonesia. Confirmed TB cases (CS, 105), symptomatic confirmed non-TB controls (CN1, 104), and asymptomatic controls (CN2, 102) were recruited at primary health centres from August to December 2024. Inclusion criteria were informed consent and ability to communicate; controls with a history of active TB were excluded from analysis. Participants were interviewed regarding individual characteristics, and a home visit made to record household conditions (temperature, humidity, carbon dioxide content and wind speed of living room air, house dimensions and construction materials). Multivariable multinomial logistic regression identified associations (relative probability ratios) of individual and household variables between cases and each control group.

**Results:**

Case status was associated with small bedroom volume (< 12m^3^), compared with CN2 (3.74 [1.25, 11.20]), and with gypsum/plaster ceilings compared with CN1 (11.24 [2.52, 50.10]. In both comparisons, case status was associated with outdoor occupation (10.13, [1.69, 60.61] and 18.60, [2.53, 137.0]), frequent coffee shop visits (10.17 [1.32, 78.55] and 24.06 [2.22, 261.0]), and time elapsed of > 1 year since the prior TB patient (21.64 [8.73, 53.64] and 13.91 [5.82, 33.24]). Compared with CN1, case status was associated with the prior patient being a sibling (8.72 [2.33, 32.57]), grandparent/parent (5.31 [1.51, 18.70]) or other relative (4.23 [1.16, 15.45]) rather than spouse. The relative probability of outdoor activity was lower among cases (0.25, [0.10, 0.62] compared with CN2), and that of being underweight higher (4.71 [1.77, 12.53] and 6.35 [2.35, 17.18]), possibly consequences of ill-health.

**Conclusion:**

Among this population, small bedroom volume, gypsum/plaster ceiling, frequent coffee shop visits, outdoor occupation, non-spousal relationship with the prior TB patient and elapsed time of > 1 year increased the relative probability of being a TB patient.

These findings suggest interventions such as improving bedroom space and targeted screening in households with prior TB, especially after one year. Housing improvements, though long-term, may usefully complement behavioural interventions for sustainable TB control.

**Supplementary Information:**

The online version contains supplementary material available at https://doi.org/10.1186/s12890-026-04434-5.

## Introduction

Global Tuberculosis report 2025 data indicate that two-thirds of the global total TB came from 8 countries: India (25%), Indonesia (10%), the Philippines (6.8%), China (6.5%), Pakistan (6.3%), Nigeria (4.8%), the Democratic Republic of the Congo (3.9%) and Bangladesh (3.6%). These patients were more commonly male (54%), and 11% were children below 14 years old. Indonesia has been among the top 3 countries ranked by the global burden of TB disease in recent years [1].

Globally, in 2024, the number of people who were newly diagnosed with TB was around 131 cases per 100,000 population [1]. In Indonesia – a low to middle income country – the annual incidence in 2024 was 382 cases per 100,000 population [2]. These high numbers of cases and the high population density coupled with difficult geographical conditions, were reported to have a significant impact on TB management in Indonesia [3]. Specifically, in West Sumatra, the annual incidence in 2022 was 245 cases per 100,000 population, and in Padang City, 228 cases per 100,000 population [4]. In such high-incidence settings, household exposure plays an important role in TB transmission to other household members [5] and may influence the development of active TB.

Various individual and environmental factors have been reported to be associated with pulmonary TB. Individual host and behavioural factors that increase vulnerability to developing TB include younger age [6, 7], being male [8], poverty [9], having an outdoor job [10], having low education [9], undernutrition [11, 12]) and behavioural exposures such as smoking [13] and social activities involving close contact with others [14, 15].

Similarly, the risk of developing pulmonary TB has been reported to be higher under environmental conditions such as overcrowding and poor ventilation through the mechanism of increased airborne transmission. Elevated CO_2_ levels, often the result of poor ventilation, as well as high levels of PM2.5, are reported to possibly disrupt immune regulation, weakening the body’s ability to contain latent infection [16]. High levels of CO_2_ have also been reported to increase the virulence of MTB [9]. Humidity [17], temperature [17], and dust [18] from building materials further shape bacterial survival and persistence indoors [19]. Together, these factors highlight how household and environmental quality can contribute to TB risk, complementing host and behavioural determinants.

In summary, although previous studies have examined a wide range of TB risk factors, including host-related risks, behavioural exposures and environmental parameters, most have focused on socioeconomic status or general housing quality. These factors may overlap and confound one another and should be considered together.

This study aims to assess how house dimensions, ventilation and building materials, in conjunction with individual vulnerability and family level factors, contribute to the risk of active TB among individuals living in households with a prior TB patient in Padang, West Sumatra, a city where TB incidence is twice the global average. By addressing household, individual and family characteristics at the same time, this research aims to inform local strategies for TB prevention and care strategies in this endemic area.

## Material and methods

### Study setting

The study was conducted in Padang City, the capital and largest city of West Sumatra Province, Indonesia. The total population of Padang in 2024 was 942,938 with an area of 694.96 km^2^. The Primary Health Care Centre (Puskesmas) is a technical implementation unit of regency health that has primary functions as a first-level health care provider, including TB prevention and care among the community [20]. Padang has 24 Puskesmas, from which 6 with high TB incidence were selected for this study, namely those in Andalas, Alai, Kuranji, Pauh, Rawang, and Nanggalo.

### Study design and population

The study was designed a case–control study with two types of control. The source population was patients, male or female, of any age, including children, who visited any of the selected Puskesmas during the study period, August to December 2024, and resided in a house where a previous TB patient had lived. A case in this study was a newly diagnosed TB patient of any age diagnosed within the previous 1 month and the home in which he/she resided.

We included two control groups to examine whether household conditions were specifically associated with active pulmonary TB, rather than with respiratory symptoms in general. Control Group 1 (CN1) comprised symptomatic TB-negative individuals, allowing us to test whether household exposures were associated with TB independently of general respiratory symptoms. Control Group 2 (CN2) comprised individuals attending the Puskesmas but without respiratory symptoms, thereby providing a comparison between cases and asymptomatic community controls. This design tested two distinct hypotheses: (1) that household risk factors differ between TB and other respiratory conditions, and (2) that they differ between TB and asymptomatic status.

Both cases and controls were confirmed to have had an earlier TB case in their respective household, with time since the earlier TB diagnosis recorded. Characteristics at the individual and household levels were compared between the groups.

### Recruitment procedures

Participants were recruited through direct contact at the Puskesmas, or via telephone contact based on patient records at the Puskesmas, by trained research staff in collaboration with TB programme officers.

### Participant inclusion criteria

For all participants, the initial inclusion criteria were 1. visiting one of the selected Puskesmas, 2. reported to have had a previous TB case in the home (via the Indonesian National Tuberculosis System records, TB staff consultation, direct interviews, or screening), and 3. willing to participate. Only one participant from any one household was included.

A specific inclusion criterion for both cases and controls 1 was undergoing diagnostic testing for TB at the Respiratory Department of the selected Puskesmas. For cases, the diagnosis of pulmonary TB needed to be confirmed by Xpert Mycobacterium tuberculosis/Rifampicin (Xpert MTB/RIF), Acid-Fast Bacilli (AFB) smear, or chest X-ray, and for controls 1 a negative result on Xpert MTB/RIF was needed.

For control-2 participants the inclusion criterion was attendance at a Puskesmas for general health concerns without any respiratory symptoms. They were screened for absence of respiratory symptoms and having had a TB patient in the house in the past using a brief questionnaire administered by admission staff or the research team. However, CN2 did not undergo any test for TB, and neither CN1 nor CN2 underwent tuberculin skin test or interferon gamma release assay (IGRA) to exclude latent TB infection (LTBI).

### Exclusion criteria

**Fig. 1:**
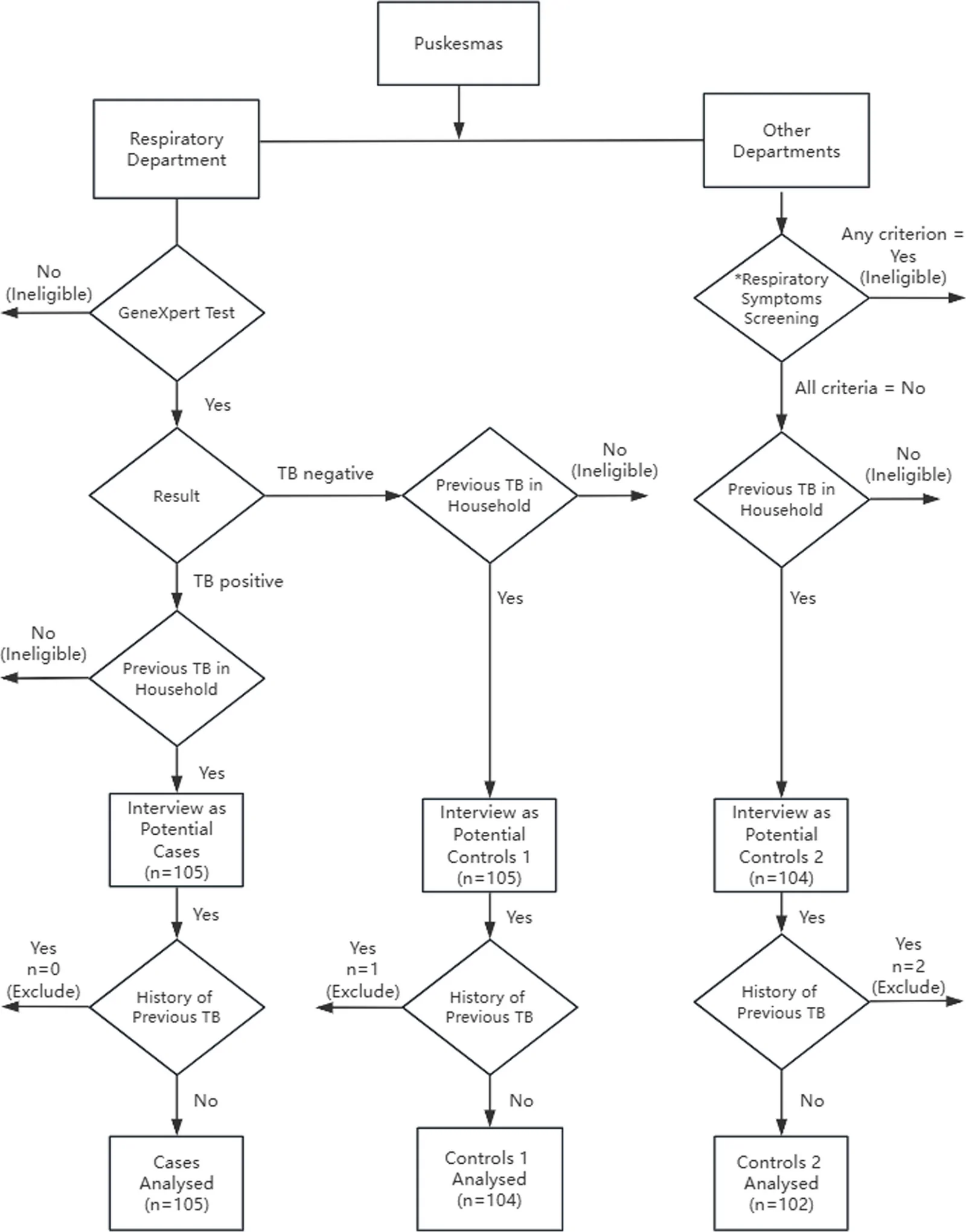
Participant flow. *Respiratory symptoms comprise: 1. Persistent cough within the previous 2 weeks; 2. Fever within the previous 2 weeks; 3. Weight loss over the previous 3 months

Individuals who could not communicate effectively during interview using the initial or main questionnaire based on the researcher's observation or inability to complete the interview were excluded. No specific diagnostic measurement for inability to communicate was used. In addition, those who revealed in the questionnaire conducted at the Puskesmas or their home that they had previously had active TB were excluded from the analysis. Participant flow is shown in Fig. 1.

### Data collection and variables

Participants were accessed at the time of visiting the Puskesmas or through the recent (previous month) Puskesmas records. After confirming eligibility, participants received a verbal explanation of the study and were asked to provide written informed consent. Where possible, consenting participants were interviewed directly at the Puskesmas visit. Those patients who could not be interviewed at the time of Puskesmas visit were interviewed during the subsequent home visit arranged according to the participant’s availability. The interviews were conducted using structured face-to-face questionnaires.

The main questionnaire was developed specifically for this study, but with the part on thermal comfort modified from the American Society of Heating, Refrigeration and Air-Conditioning Engineers Standard 55–2017 [21, 22]. Questions on cooking fuel use and household structure items were modified from the Indonesian Health Survey (SDKI) [23] and social activity measures from a previous study related to travel and social activity among TB patients [24]. The combined instrument was pilot tested with five households in Simabur Village, West Sumatra, a setting similar to that of the study population. Based on pilot feedback, questions were refined for clarity before use. The interview was conducted in the Indonesian language. The English version of the questionnaire is included as Supplementary File 1.

The interview collected data on individual sociodemographic characteristics, vulnerability factors, external vulnerability and known TB exposure history. Individual-level sociodemographic variables included sex, age, marital status, occupation, religion, education, BMI and morbidities. Family-level variables included income, type of cooking fuel and presence of smokers in the home. External vulnerability was assessed by asking about daily visits to coffee shops, and visiting other social places such as local facilities or playing outdoor sports. Smoking was recorded separately for individual history and household exposure. Characteristics of previous TB patients in the household included time since diagnosis, relationship with the study participant, type of TB, and outcome.

A home visit was arranged for each participant to assess household conditions. Contact details obtained during the interviews were used to coordinate the visits. Household conditions were evaluated through direct observation and a checklist, and the questionnaire, if not already completed, also administered to the participant during the visit.

### Household conditions

Data on household conditions included housing type (apartment, traditional or modern), room level assessments such as bedroom (of the enrolled participant) and living room dimensions, usual number of occupants of living room and bedroom, number of windows in the bedroom and living room, materials used for construction of floor, wall, living room ceiling and roof, household smoking, ventilation (typical window opening practices for the past 1 year) and signs of dampness.

Environmental measurements were conducted during daytime hours (08:00–17:00) in the living room at each household visit. Ambient temperature and relative humidity were measured using a Peak Meter PM6508 Digital Temperature and Humidity Meter (temperature range −20 to 60 °C, precision ± 0.5 °C; humidity range 0–100% RH, accuracy ± 2.0% RH). Carbon dioxide (CO₂) concentration was measured as an indicator of ventilation using a Sndway SW723 Carbon Dioxide Detector (range: 0–9999 ppm, resolution: 1 ppm). All readings were recorded after the digital display stabilized (3–5 s).

Air speed was measured using a handheld anemometer digital wind speed meter (GM816 Pocket LCD Digital Anemometer, with a range of 0−30 m/s and accuracy of ± 5%). Measurement was performed at each visit using a portable mini-fan (Model: RT-BF25) to generate a low-speed reference airflow, and natural air speed was then measured and interpreted relative to this reference. All instruments were held at chest level (approximately 1.2–1.3 m). Windows and doors were left in their current state.

Room volume was calculated from tape-measured dimensions, and building materials were documented. Measurements were taken once per household visit. This one-time measurement was used as a proxy for exposure.

### Data management

All data were recorded using Kobo Toolbox [25]. The final review for data cleaning, missing data, and error checking was done using Stata release 18 [26].

### Statistical analysis

Housing conditions were initially compared across the 3 groups using tabulation. For several continuous parameters of housing condition (room volume, number of persons, CO_2_ level), the extreme quartile (poorest condition) was compared against the remaining conditions. Housing structure was grouped into five categories based on the most common combinations of wall, roof and ceiling materials.

**Fig. 2:**
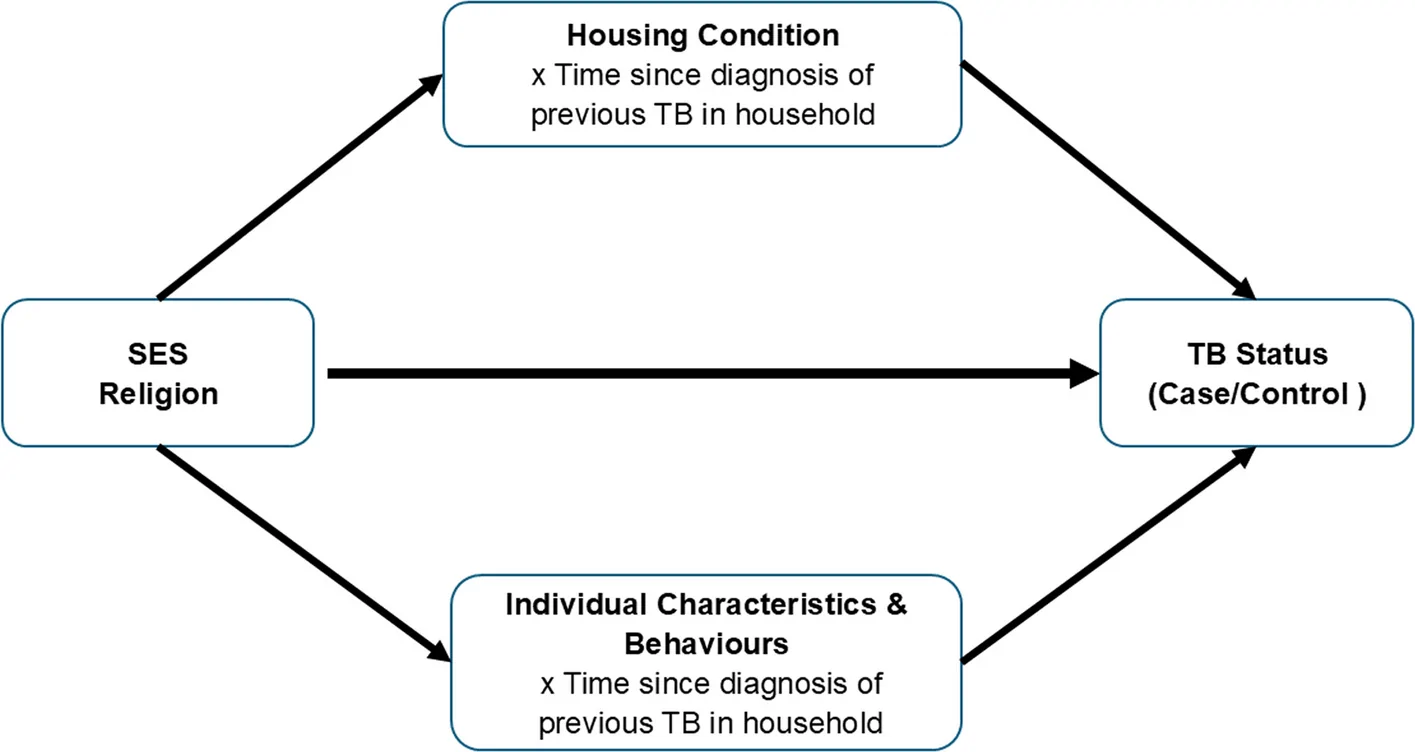
Conceptual framework

Associations between case/control status and household conditions and individual characteristics were explored based on the conceptual framework depicted in Fig. 2. In this model, the arrows show the hypothesized direction of influence. Thus, for the association of outcome (case/control status) with main exposures of interest (housing condition and individual characteristics), socio-economic status (SES) and religion were considered to be potential confounders. Time since diagnosis of the previous TB case in the household was considered to potentially modify the association between outcome and both individual and household exposures.

The questionnaire items on coffee shop visits and social activities are provided in Supplementary File 1 (Section D). Initially, coffee shop visits were recorded in four frequency categories (daily ≥ 30 min, weekly, less than weekly, never). After reviewing their distribution, they were combined into two groups (never/rarely vs. daily) to ensure sufficient sample size and analytical clarity.

Following cross tabulation, univariable multinomial logistic regression models of household condition were constructed. Selection of variables to represent household condition and individual characteristics were initially selected based on their showing a bivariate association with case–control status with a p-value of ≤ 0.2. Selected variables were included in a series of multivariable multinomial models, which were then refined by sequential removal of least (and non-significant) variables as revealed by likelihood ratio (LR) tests. An overall likelihood ratio p-value (LR p) of ≤ 0.05 was considered statistically significant in the multivariable model. Decision on the removal or retention of variables with borderline significance was aided by consideration of the Akaike Information criterion (AIC). The magnitude of associations was identified from the relative probability ratio (RPR). Likelihood ratio p-values for each comparison (case versus control 1 and case versus control 2) were determined by applying appropriate constraints to the models.

At first, separate multivariable models were constructed for household conditions alone with the addition of common potential confounder variables (household income and family religion). The same sequence was followed for individual characteristics, and, finally, the two sets of variables (household and individual) were included together. All analyses were conducted using Stata, release 18 [26].

### Sample size calculation

The required sample size for each group was based on a formula comparing exposure proportions between two groups, as the prespecified hypotheses being tested separately compared cases with each of the controls. To detect an odds ratio of ≥ 2.5 for residing in poor housing conditions [27] with 80% power and type I error of 0.05, with the prevalence of poor condition in control households of between 20 and 60%, a sample size of 105 individuals per group was estimated to be adequate. This sample size would be sufficient to detect an odds ratio of 1.8 when comparing exposures to the overall most extreme quartile of initially continuous variables.

### Ethical approval

The study was approved by the Padang City Government Office, as documented in EC Number: 070.11749/DPMPTSPPP/VIII/2024. Written informed consent was requested from all potential respondents before their participation. Only after potential participants gave documented consent was the interview conducted. Computerized data did not indicate the identity of any participant.

## Results

### Household conditions and participant characteristics

In the questionnaire-based interview, 3 participants, one among the control-1 group and two in the control-2 group, revealed a history of active TB and were excluded from subsequent analysis. Thus, the analysed sample comprised 311 participants: 105 TB cases, 104 controls 1, and 102 controls 2. Among controls 2, the reasons for visiting the Puskesmas varied, but the absence of respiratory symptoms was confirmed by interview in all.

Household conditions among each group of participants are shown in Table 1. Overall, the participants’ houses were mostly attached, of non-traditional (or modern) design, one-storey and built with the lowest storey at ground level. Common materials for floor, walls, roof and ceiling were ceramic, cement, metal sheet and wood, respectively. Depending on the structural combinations, 5 categories of house construction were identified, the most common combination being cement walls, metal roof and wooden ceilings (60.1%) followed by wooden walls, metal roof and wooden ceiling (15.8%) (Table 1).

**Table 1:** Household conditions by participant group

Variable	Case (*n =* 105)	Control 1 (*n =* 104)		Control 2 (*n =* 102)	*P-*value^**a**^	Unadjusted RPR (95% CI)				LR P-value^**c**^
						Case vs CN1	*P*-value^**b**^	Case vs CN2	*P*-value^**b**^	
House type					.220					.205
Traditional	20 (19.1)	12 (11.5)		20 (19.6)		Ref		Ref		
Modern	85 (80.9)	92 (88.5)		82 (80.4)		0.55 (0.26, 1.20)	.135	1.04 (0.52, 2.07)	.919	
House attached/detached					.037					.022
Attached	96 (91.4)	103 (99.0)		97 (95.1)		Ref		Ref		
Detached	9 (8.6)	1 (1.0)		5 (4.9)		9.66 (1.20, 77.65)	0.033	1.82 (0.59, 5.62)	0.299	
House storeys					.280					.299
1 storey	99 (94.3)	98 (94.2)		91 (89.2)		Ref		Ref		
2 storeys	6 (5.7)	6 (5.8)		11 (10.8)		0.99 (0.31, 3.18)	.986	0.50 (0.18, 1.41)	.191	
Floor elevation					.611		.324		.870	.558
On ground	88 (83.8)	95 (91.4)		86 (84.3)		Ref		Ref		
1 m	9 (8.6)	6 (5.8)		10 (9.8)		1.62 (0.55, 4.73)	.379	0.88 (0.34, 2.27)	.791	
2 m	4 (3.8)	1 (1.0)		4 (3.9)		4.32 (0.47, 39.38)	.195	0.98 (0.24, 4.03)	.975	
3 m	4 (3.8)	2 (1.9)		2 (2.0)		2.16 (0.39, 12.08)	.381	1.95 (0.35, 10.95)	.446	
House orientation (front-facing direction)					.931		.682		.857	
North	11 (10.5)	16 (15.4)		16 (15.7)		Ref		Ref		.943
Northeast	8 (7.6)	11 (10.6)		10 (9.8)		1.06 (0.32, 3.48)	.926	1.16 (0.35, 3.89)	.805	
East	20 (19.0)	15 (14.4)		17 (16.7)		1.66 (0.58, 4.75)	.343	1.55 (0.55, 4.40)	.409	
Southeast	13 (12.4)	11 (10.6)		7 (6.9)		1.45 (0.47, 4.52)	.518	2.29 (0.68, 7.74)	.184	
South	17 (16.2)	16 (15.4)		16 (15.7)		1.55 (0.55, 4.32)	.406	1.55 (0.55, 4.40)	.406	
Southwest	12 (11.4)	9 (8.7)		11 (10.8)		1.94 (0.61, 6.16)	.261	1.59 (0.52, 4.87)	.420	
West	14 (13.3)	20 (19.2)		15 (14.7)		0.84 (0.29, 2.45)	.752	1.07 (0.36, 3.18)	.908	
Northwest	10 (9.5)	6 (5.8)		10 (9.8)		2.42 (0.68, 8.64)	.172	1.45 (0.45, 4.66)	.529	
Roof material					.034					.024
Tile/asphalt/+	7 (6.7)	2 (1.9)		11 (10.8)		Ref		Ref		
Metal	98 (93.3)	102 (98.1)		91 (89.2)		0.27 (0.06,1.35)	.112	1.69 (0.63, 4.55)	.297	
Ceiling material					.029		.002		.456	.016
Wood	74 (70.5)	88 (84.6)		79 (77.5)		Ref		Ref		
Gypsum/plaster	17 (16.2)	3 (2.9)		11 10.8)		6.74 (1.90, 28.89)	.003	1.65 (0.73, 3.75)	,233	
Other	14 (13.3)	13 12.5)		12 (11.8)		1.28 (0.57, 2.90)	.552	1.25 (0.54, 2.97)	.606	
Floor material					.470		.586		.661	.452
Cement	36 (34.3)	38 (36.5)		32 (31.4)		Ref		Ref		
Wood/other	6 (5.7)	3 (2.9)		9 (8.8)		2.11 (0.49, 9.08)	.315	0.59 (0.19, 1.85)	.367	
Ceramic	63 (60.0)	63 (60.6)		61 (59.8)		1.06 (0.59, 1.87)	.854	0.92 (0.51, 1.66)	.777	
Wall material					.648		.660		.621	.650
Cement	80 (76.2)	83 (79.8)		73 (71.6)		Ref		Ref		
Wood	21 (20.0)	19 (18.3)		26 (25.5)		1.15 (0.57, 2.29)	.698	0.74 (0.38, 1.42)	.362	
Ceramic/other	4 (3.8)	2 (1.9)		3 (2.9)		2.08 (0.37, 11.65)	.407	1.22 (0.26, 5.62)	.802	
House construction category *					.145		.094		0.312	.139
1	60 (57.1)	72 (69.2)		55 (53.9)		Ref		Ref		
2	13 (12.4)	14 (13.5)		22 (21.6)		1.10 (0.49, 2.6)	.122	0.54 (0.25, 1.18)	.798	
3	10 (9.5)	3 (3.9)		6 (5.9)		4.00 (1.1, 15.2)	.440	1.53 (0.52, 4.48))	.042	
4	5 (4.8)	4 (3.9)		2 (2.0)		1.50 (0.39, 5.84)	.333	2.29 (0.43, 12.30)	.559	
5	19 (16.8)	11 (10.6)		17 (16.7)		1.86 (0.81, 4.23)	.824	0.92 (0.43, 1.97)	.146	
Household size (persons)					.776		.362		.973	.779
< 3	15 (14.3)	11 (10.6)		13 (12.8)		Ref		Ref		
3–4	34 (32.4)	44 (42.3)		34 (33.3)		0.57 (0.23, 1.39)	.215	0.87 (0.36, 2.09)	.750	
5–6	38 (36.2)	32 (30.8)		39 (38.2)		0.87 (0.35, 2.16)	.766	0.84 (0.35, 2.01)	.702	
≥ 7	18 (17.1)	17 (16.3)		16 (15.7)		0.78 (0.28, 2.16)	.628	0.97 (0.36, 2.66)	.961	
Bedroom volume (m^3^)					.001					.001
≥ 12	73 (69.5)	87 (83.7)		91 (89.2)		Ref		Ref		
< 12	32 (30.5)	17 (16.3)		11 (10.8)		2.24 (1.15, 4.36)	.017	3.63 (1.71, 7.69)	.001	
Usual number of people sleeping in the bedroom					.510					.515
< 3	72 (68.6)	78 (75.0)		76 (74.5)		Ref		Ref		
≥ 3	33 (31.4)	26 (25.0)		26 (25.5)		1.38 (0.75, 2.51)	.303	1.34 (0.73, 2.46)	.345	
Bedroom windows opened					.029					.028
Yes	61 (58.1)	68 (65.4)		77 (75.5)		Ref		Ref		
Rarely/none	44 (41.9)	36 (34.6)		25 (24.5)		1.36 (0.78, 2.38)	.279	2.22 (1.23, 4.03)	.009	
Living room volume (m^3^)					.022					.021
≥ 9	70 (66.7)	78 (75.0)		85 (83.3)		Ref		Ref		
< 9	35 (33.3)	26 (25.0)		17 (16.7)		1.50 (0.82, 2.74)	.186	2.50 (1.29, 4.84)	.007	
Usual number of persons in the living room					.164					.138
≤ 3	91 (86.7)		91 (87.5)	96 (94.1)		Ref		Ref		
> 3	14 (13.3)		13 (12.5)	6 (5.9)		1.08 (0.48, 2.42)	.857	2.46 (0.91, 6.68)	.077	
Living room vol. per person (m^3^/person)					.002					.001
≥ 4	68 (64.8)	76 (73.1)		88 (86.3)		Ref		Ref		
4	37 (35.2)	28 (26.9)		14 (13.7)		1.48 (0.82, 2.66)	.195	3.42 (1.71, 6.83)	<.001	
Livingroom windows opened					.442					.435
Yes	93 (88.6)	88 (84.6)		84 (82.4)		Ref		Ref		
Rarely/none	12 (11.4)	16 (15.4)		18 (17.6)		0.71 (0.32, 1.58)	.403	0.60 (0.27, 1.32)	.207	
Smoking in household					.598					.602
No	29 (27.6)	29 (27.9)		34 (33.3)		Ref				
Yes	76 (72.4)	75 (72.1)		68 (66.7)		1.02 (0.55,1.86)	.214	1.31 (0.72, 2.40)	.103	
Pet type					.177		.478		.512	.644
No pet	74 (70.5)	78 (75.0)		70 (68.6)		Ref		Ref		
Chicken	9 (8.6)	2 (1.9)		4 (3.9)		2.64 (0.50, 14.00)	.256	1.58 (0.36, 6.85)	.543	
Cat/dog	22 (20.9)	24 (23.1)		28 (27.5)		0.97 (0.50, 1.87)	.919	0.74 (0.39, 1.42)	.369	
Handwashing facility type					.328					.330
Bucket/dipper	36 (34.3)		26 (25.0)	29 (28.4)		Ref		Ref		
Sink/tap-water/gallon	69 (65.7)		78 (75.0)	73 (71.6)		0.64 (0.35, 1.16)	.143	0.76 (0.42, 1.37)	.365	
Clean water for handwashing					-					-
Yes	105 (100.0)	104 (100.0)		102 (100.0)		-	-	-		
Soap availability					.599					-
No	0 (0.0)	1 (1.0)		1 (1.0)						
Yes	105 (100.0)	103 (99.0)		101 (99.0)		-	-	-	-	
Handwashing regularly						-	-	-	-	
Yes	105 (100.0)	104 (100.0)		102 (100.0)	-					
Waste bin inside house					.030					.017
Open	91 (86.7)	100 (96.2)		88 (86.3)		Ref		Ref		
Closed	14 (13.3)	4 (3.9)		14 (13.7)		3.85 (1.22, 12.11)	.021	0.97 (0.44, 2.14)	0.934	
Waste disposal					.040		.129		.125	.047
Transported	10 (9.5)	9 (8.7)		19 (18.6)		Ref		Ref		
Throw away	65 (61.9)	77 (74.0)		61 (59.8)		0.76 (0.29, 1.98)	.574	2.02 (0.87, 4.70)	.100	
Burned	30 (28.6)	18 (17.3)		22 (21.6)		1.50 (0.51, 4.39)	.459	2.59 (1.01, 6.65)	.048	
Cooking fuel type					.158					.137
Clean fuel	97 (92.4)	102 (98.1)		97 (95.1)		Ref		Ref		
Dirty fuel	8 (7.6)	2 (1.9)		5 (4.9)		4.21 (0.87, 20.30)	.074	1.60 (0.51, 5.06)	.424	
Water leak					.492					.492
No	58 (55.2)	51 (49.0)		58 (56.9)		Ref		Ref		
Yes	47 (44.8)	53 (51.0)		44 (43.1)		0.78 (0.45, 1.34)	.370	1.07 (0.62, 1.85)	.814	
Floor dampness					.927					.928
No	93 (88.6)	91 (87.5)		91 (89.2)		Ref		Ref		
Yes	12 (11.4)	13 (12.5)		11 (10.8)		0.90 (0.39, 2.08)	.811	1.07 (0.45, 2.54)	.883	
Mould					.384					.391
No	82 (78.1)	74 (71.2)		80 (78.4)		Ref		Ref		
Yes	23 (21.9)	30 (28.8)		22 (21.6)		0.69 (0.37, 1.30)	.250	1.02 (0.53, 1.97)	.953	
CO_2_ level (ppm)					.255					.251
≤ 624	74 (70.5)	78 (75.0)		82 (80.4)		Ref		Ref		
> 624	31 (29.5)	26 (25.0)		20 (19.6)		1.26 (0.68–2.31)	.463	1.72 (0.90–3.27)	.100	
Relative Humidity (%)										
(mean ± SD)	72.34 ± 6.23	70.98 ± 6.42		71.72 ± 5.85	.284	1.02 (0.97, 1.06)	.113	1.02 (0.97, 1.06)	.476	.279
Air temperature (°C)										
(mean ± SD)	30.51 ± 2.39	31.17 ± 2.24		30.90 ± 2.19	.111	0.88 (0.78, 0.99)	.038	0.93 (0.82, 1.05)	.223	.109
Air speed (m/s)										
(mean ± SD)	0.12 ± 0.38	0.05 ± 0.26		0.10 ± 0.35	.297	2.02 (0.81, 5.05)	.131	1.16 (0.55, 2.46)	.695	.268
Monthly household income (USD)					.409		.409		.236	.415
< 153	77 (73.3)	68 (65.4)		64 (62.75)		1.13 (0.35, 3.68)	.836	2.00 (0.69, 5.82)	.200	
153–306	22 (20.9)	30 (28.8)		28 (27.5)		0.73 (0.21, 2.89)	.629	1.31 (0.41, 4.16)	.648	
> 306	6 (5.7)	6 (5.8)		10 (9.8)		Ref		Ref		
Family religion					.045					.053
Non-Islam	9 (8.6)	3 (2.9)		2 (2.0)		Ref		Ref		
Islam	96 (91.4)	101 (97.1)		100 (98.0)		0.32 (0.08, 1.21)	.092	0.21 (0.05, 1.01)	.052	

^a^Chi squared p-value

^b^Wald test p-value, (for variables with more than 2 categories the likelihood ratio test p-value is also shown)

^c^Overall likelihood ratio test p-value

^*^House construction category: 1 (cement walls, metal roofs, wood ceilings); 2 (wood walls, metal roofs, wood ceilings); 3 (cement walls, metal roofs, gypsum ceilings); 4 (wood walls, metal roofs, plaster ceilings); 5 (other combinations). Clean fuel (electricity, gas). Dirty fuel (wood, kerosene). Tile/asphalt + (tile, asphalt or other non-metal). RPR: relative probability ratio

Household monthly income was most commonly less than $153, and the religion of most families was Islam. Washing facilities were present in almost all houses. In all groups, around one-half had evidence of water leaks, with over 20% showing signs of mould (Table 1). Around 20–30% of households had a pet that was at times inside the house. Domestic waste was commonly placed in an open bin within the house before disposing of it outside. Almost all houses used clean cooking fuel, namely cooking gas or electricity.

Measurement/enquiry of room dimensions revealed a wide range of room volumes − living room from 6.8 m^3^ to 180 m^3^, and the bedroom from 7.2 m^3^ to 216 m^3^. The number of windows ranged from 0 to 9 in the bedroom and 0 to 8 in the living room. The number of people sleeping in the bedroom mostly ranged from 1 to 2, while the living room typically hosted between 1 and 3 persons (range 0 to 10).

At the time of the home visit, the indoor CO_2_ concentration varied from 417 to 973 ppm, with the top quartile comprising values > 624 ppm. Indoor air temperature ranged from 25.5 °C to 35.0 °C but for all groups the mean was around 31–32 °C, relative humidity ranged from 60 to 93% with mean in all mean groups between 70 and 73%, and air speed ranged from 0 to 1.5 m/s but with a mean in all groups of no more than 0.12 m/s.

Some difference in households was evident between the cases and both control households. Thus, case households were more likely to have a bedroom volume that was in the lowest overall quartile (< 12m^3^). Comparing cases to controls 1 and controls 2 respectively, bedroom volume < 12m^3^ showed RPR of 2.24 [1.15, 4.36] (*p* = 0.017) and 3.63 [1.71, 7.69] (*p* = 0.001).

Some other household variables showed evidence of differing between cases and controls 2 but not between cases and controls 1. For living room volume < 9m^3^, the RPR value for cases compared to controls 2 was 2.50 [1.29, 4.84] (*p* = 0.007). A similar pattern of association was seen for living room volume per person < 4m^3^ (RPR 3.42 [1.71, 6.83] *p* < 0.001, and for having no or rarely opened bedroom windows (RPR 2.22 [1.23, 4.03] *p* = 0.009. Waste was more likely to be disposed of by burning rather than by transported away by local authority (RPR 2.59 [1.01, 6.65] *p* = 0.048]).

On the other hand, case households differed from those of controls 1, but not controls 2, in being more commonly detached (RPR 9.66 [1.20, 77.65], *p* = 0.033)**,** collecting waste in a closed bin (RPR 3.85 [1.22, 12.11] *p* = 0.021)**,** and having ceilings made from gypsum or plaster rather than wood (6.74 [RPR 1.90, 28.89], *p* = 0.003). Mean room air temperature was significantly lower in case households than in control-1 households (RPR for an incremental difference of 1 Celsius degree 0.88 [0.78, 0.99] *p* = 0.038).

No significant difference between cases and controls was found for other household characteristics (number of storeys, orientation, floor and wall materials, structural combination, total number of persons in the household or number of persons usually in the living room, opening of living room windows, smoking in the household, having a pet, type of washing facilities, type of cooking fuel, water leaks or dampness, signs of mould, or high CO_2_ level, mean relative humidity, and mean air speed at the time of home visit). However, control-1 houses were relatively more likely to have a metal roof than control-2 houses (RPR 6.16 [1.33, 28.55], *p* = 0.024).

Among participant characteristics, in all groups, Muslims and having an indoor job predominated, while more than half were female, adult (mostly married), with education at high school or above. Time elapsed since the diagnosis of the previous TB patient in the home varied from < 2 months to 10 years, more likely to be over one year in cases compared with controls 1 (RPR 12.55 [6.39, 24.66], *p* < 0.001) and controls 2 (RPR 7.84 [4.20, 14.61], *p* < 0.001). Most participants in all groups reported no comorbidities. However, hypertension was significantly more common in cases compared to controls 2 (RPR 11.08 [1.39, 88.45], *p* = 0.023) (Table 2).

**Table 2:** Individual characteristics by participant group

Variable	Case (*n =* 105)	Control 1 (*n =* 104)	Control 2 (*n =* 102)	*P*-value^a^	Unadjusted RPR (95% CI)				LR *P*-value^c^
					Case vs CN1	*P*-value^b^	Case vs CN2	*P*-value^b^	
Individual characteristics									
Sex				< .001					< .001
Female	58 (55.2)	86 (82.7)	83 (81.4)		Ref		Ref		
Male	47 (44.8)	18 (17.3)	19 (18.6)		3.87 (2.05, 7.32)	< .001	3.54 (1.89, 6.64)	< .001	
Age and marital status				< .001		< .001		< .001	< .001
Youth (< 22 yrs)	36 (34.3)	17 (16.4)	10 (9.8)		Ref		Ref		
Adult unmarried	17 (16.2)	5 (4.8)	10 (9.8)		1.61 (0.51, 5.08)	.420	0.47 (0.17, 1.35)	.161	
Adult married	52 (49.5)	82 (78.8)	82 (80.4)		0.30 (0.15, 0.59)	< .001	0.18 (0.08, 0.39)	< .001	
Occupation				.002					.002
Indoor	91 (86.7)	100 (96.2)	100 (98.0)		Ref		Ref		
Outdoor	14 (13.3)	4 (3.8)	2 (2.0)		3.85 (1.22, 12.11)	.021	7.69 (1.70, 34.77)	.008	
Educational level				.688					.689
Primary & Junior School	44 (41.9)	38 (36.5)	38 (37.3)		Ref		Ref		
≥ High School	61 (58.1)	66 (63.5)	64 (62.8)		0.80 (0.46, 1.39)	.427	0.82 (0.47, 1.44)	.494	
BMI				< .001		< .001		< .001	< .001
Underweight	51 (48.6)	13 (12.5)	10 (9.8)		4.43 (2.14, 9.57)	< .001	5.88 (2.68, 12.88)	< .001	
Normal	46 (43.8)	52 (50.0)	53 (52.0)		Ref		Ref		
Overweight/obese	8 (7.6)	39 (37.5)	39 (38.2)		0.23 (0.10, 0.55)	.001	0.24 (0.10, 0.56)	.001	
Comorbidity				.083					.052
No disease	92 (87.6)	90 (86.5)	83 (81.4)		Ref		Ref		
Diabetes Mellitus	7 (6.7)	6 (5.8)	8 (7.8)		0.88 (0.28, 2.71)	.818	1.27 (0.44, 3.65)	.661	
Hypertension	1 (1.0)	6 (5.8)	10 (9.8)		6.13 (0.72, 51.96)	.096	11.08 (1.39, 88.45)	.023	
Other Disease	6 (4.76)	2(1.9)	1 (1.0)		0.41 (0.08, 2.16)	.293	0.22 (0.03, 1.94)	.173	
Smoking history				.001					.001
No	80 (76.2)	97 (93.3)	91 (89.2)		Ref		Ref		
Yes	25 (23.8)	7 (6.7)	11 (10.8)		4.33 (1.78-, 10.53)	.001	2.59 (1.20, 5.58)	.016	
Coffee shop visit				.001					.002
Never/rarely	94 (89.5)	102 (98.1)	101 (99.0)		Ref		Ref		
Daily for 30 min	11 (10.5)	2 (1.9)	1 (1.0)		5.97 (1.29, 27.63)	.022	11.82 (1.50, 93.32)	.019	
Visit social place				< .001					< .001
Never/rarely	46 (43.8)	25 (24.0)	17 (16.7)		Ref		Ref		
Outdoor activity /sport/local facility	59 (56.2)	79 (76.0)	85 (83.3)		0.41 (0.22, 0.73)	.003	0.26 (0.13, 0.49)	< .001	
Previous TB patient in household									
Time since diagnosis				< .001		< .001		< .001	< .001
≤ 1 year	32 (30.5)	88 (84.6)	79 (77.4)		Ref		Ref		
> 1 year	73 (69.5)	16 (15.4)	23 (22.6)		12.55 (6.39, 24.66)		7.84 (4.20, 14.61)		
Relationship with participant				< .001		< .001		.006	< .001
Spouse	8 (7.6)	35 (33.7)	24 (23.5)		Ref		Ref		
Sibling	29 (27.6)	17 (16.3)	20 (19.6)		7.46 (2.82, 19.76)	< .001	4.35 (1.63, 11.62)	.003	
Parent/grandparent	23 (21.9)	31 (29.8)	26 (25.5)		3.25 (1.27, 8.30)	.014	2.65 (1.00, 7.05)	.050	
Other	45 (42.9)	21 (20.2)	32 (31.4)		9.38 (3.71, 23.68)	< .001	4.22 (1.68, 10.58)	.002	
Type of TB				.170					.184
Non-MDR	104 (99.0)	102 (98.1)	99 (97.1)		Ref		Ref		
MDR	1 (1.0)	2 (1.9)	3 (2.9)		0.49 (0.04, 5.49)	.563	0.19 (0.02, 1.63)	.128	
Outcome				< .001		< .001		< .001	< .001
Cured/completed	75 (71.4)	44 (42.3)	49 (48.0)		Ref		Ref		
Relapsed	7 (6.7)	2 (1.9)	5 (4.9)		2.05 (0.41, 10.32)	.383	0.91 (0.27, 3.05)	.884	
Died	4 (3.8)	3 (2.9)	6 (5.9)		0.78 (0.17, 3.66)	.755	0.44 (0.12, 1.62)	.216	
LTFU	5 (4.8)	4 (3.8)	1 (1.0)		0.73 (0.19, 2.88)	.656	3.27 (0.37, 28.81)	.287	
On treatment	14 (13.3)	51 (49.0)	41 (40.2)		0.16 (0.08, 0.32)	< .001	0.22 (0.11, 0.45)	< .001	

Outdoor occupation (agriculture / fishery, labour/ manual work). Indoor occupation (student/ retired / homemaker / housewife / civil servant / state enterprise). BMI categories underweight (< 18.5 kg/m^2^), normal (18.50–24.99 kg/m^2^), overweight/ obese (≥ 25 kg/m^2^). Other disease (chronic kidney disease, asthma, other chronic disease)

RPR: relative probability ratio. CI: confidence interval. MDR: multidrug resistant

^a^Chi squared p-value

^b^Wald test p-value, (for variables with more than 2 categories the likelihood ratio test p-value is also shown)

^c^Overall likelihood ratio test p-value

Males, age younger than 22 years compared with married adults, having an outdoor occupation, a history of smoking, being underweight, and spending time in coffee shops, were more common in cases than in controls 1 and controls 2. However, cases less commonly reported going outside to other social places, such as local facilities or playing sports (Table 2).

Compared with controls, the relationship of the earlier TB patient in the household to the study participant was more commonly sibling or parent/grandparent and less commonly a spouse among cases, and the earlier patient was less likely to be currently on treatment than to have completed treatment or been cured (Table 2).

### Multivariable models

Prior to multivariable modelling, the likely correlations among several household conditions, such as living room volume, bedroom volume, number of persons in the household, usual number in the living room and living room volume per person were explored. These relationships were considered in constructing and interpreting the models (Models 1, 2 and 3, Tables 3 and 4). Candidate variables for initial inclusion in the multivariable model comprised those having an overall likelihood ratio p-value in the univariable analysis of less than 0.2, even though they may have shown no evidence in univariable analysis of an association between cases and one or other of the control groups. Several variables which had a significant univariable association with case–control status showed weaker and/or non-significant association in the multivariable environment. Living room and bedroom volumes were moderately correlated (*r* = 0.442, *p* < 0.001) and strongly associated when dichotomized into the lowest quartile or above (*p* < 0.001, chi square test). Based on the AIC of the separate models with, respectively, bedroom volume and living room volume, inclusion of bedroom volume resulted in lower AIC value, and therefore was selected over living room volume in the multivariable models. The variables of monthly household income and religion were included throughout the modelling as they were considered to represent potential confounding conditions.

**Table 3:** Multivariable analysis among CS vs CN1

Variable	CS VS CN1					
	Model 1		Model 2		Model 3	
	RPR (95% CI)	LR- p	RPR (95% CI)	LR- p	RPR (95% CI)	LR- p
Household conditions						
Bedroom vol (m^3^) (ref: ≥ 12)		.105				.106
< 12	2.00 (0.86, 4.64)				2.40 (0.83, 6.99)	
Ceiling material (ref: wood)		<.001				.002
Gypsum/Plaster	13.62 (3.43, 54.18)				11.24 (2.52, 50.10)	
Other	1.47 (0.55, 3.92)				1.26 (0.36, 4.49)	
Time elapsed since the previous patient ref: (< 1 year)		<.001		<.001		<.001
> 1 year	15.39 (7.44, 31.84)		19.38 (8.17, 45.99)		21.64 (8.73, 53.64)	
Common confounders						
Monthly household income (USD) (ref: > 306)		.588		.403		.680
< 153	0.65 (0.16, 2.59)		0.35 (0.08, 1.52)		0.51 (0.11, 2.37)	
153–306	0.49 (0.11, 2.12)		0.39 (0.08, 1.88)		0.60 (0.12, 3.08)	
Religion (re: non-Muslim)		.192				.665
Islam	0.35 (0.07, 1.75)		0.50 (0.08, 3.19)	.157	0.65 (0.09, 4.75)	
Individual characteristics						
Occupation (ref: indoor)				.012		.008
Outdoor			7.51 (1.43, 38.45)		10.13 (1.69, 60.61)	
BMI (ref: normal)				<.001		<.001
Underweight			4.69 (1.83, 12.04)		4.71 (1.77, 12.53)	
Overweight/obese			0.30 (0.11, 0.84)		0.36 (0.12, 1.05)	
Relationship (ref: spouse)				.002		.006
Sibling			9.70 (2.67, 35.19)		8.72 (2.33, 32.57)	
Parent/grandparent			5.66 (1.64, 19.56)		5.31 (1.51, 18.70)	
Other			4.33 (1.20, 15.62)		4.23 (1.16, 15.45)	
Coffee shop visit frequency (ref: never/rarely)				.008		.012
Daily for 30 min			10.41 (1.44, 75.48)		10.17 (1.32, 78.55)	
Visit social place (ref: never/rarely)				.083		.066
Outdoor activity/sport/local facility			0.46 (0.20, 1.11)		0.43 (0.17, 1.06)	
AIC	604.3503		543.5616		538.4052	

Model 1. Household conditions adjusted for common confounders

Model 2. Individual characteristics adjusted for common confounders

Model 3. Household conditions and individual characteristics adjusted for common confounders

*RPR* Relative probability ratio, *LR-p* Likelihood ratio test *p*-value

**Table 4:** Multivariable analysis among CS vs CN2

Variable	CS VS CN2					
	Model 1		Model 2		Model 3	
	RPR (95% CI)	LR- p	RPR (95% CI)	LR- p	RPR (95% CI)	LR- p
Household conditions						
Bedroom vol (m^3^) (ref: ≥ 12)		.012				.016
< 12	3.03 (1.25, 7.33)				3.74 (1.25, 11.20)	
Ceiling material (ref: wood)		.032				.201
Gypsum/Plaster	3.56 (1.35, 9.40)				2.83 (0.90, 8.87)	
Other	1.31 (0.50, 3.47)				1.10 (0.31, 3.89)	
Time elapsed since the previous patient (ref: < 1 year)		<.001				<.001
> 1 year	8.62 (4.39, 16.92)		12.94 (5.66, 29.60)	<.001	13.91 (5.82, 33.24)	
Common confounders						
Monthly household income (USD) (ref: > 306)		.847		.814		.803
< 153	1.04 (0.30, 3.56)		0.65 (0.16, 2.56)		0.73 (0.17, 3.08)	
153–306	0.83 (0.22, 3.11)		0.73 (0.16, 3.25)		0.95 (0.20, 4.42)	
Religion (ref: non-Muslim)		.203		.157		.439
Islam	0.33 (0.06, 1.98)		0.25 (0.03, 1.92)		0.42 (0.05, 3.93)	
Individual characteristics						
Occupation (ref: indoor)				.003		.001
Outdoor			13.08 (1.99, 85.33)		18.60 (2.53, 137.02)	
BMI (ref: normal)				<.001		<.001
Underweight			5.65 (2.14, 14.88)		6.35 (2.35, 17.18)	
Overweight/obese			0.28 (0.10, 0.77)		0.31 (0.11, 0.88)	
Relationship (ref: spouse)				.049		.107
Sibling			5.34 (1.47, 19.39)		4.57 (1.23, 16.93)	
Parent/Grandparent			2.31 (0.67, 7.95)		2.10 (0.61, 7.29)	
Other			3.36 (0.91, 12.43)		2.95 (0.80, 10.92)	
Coffee shop visit frequency (ref: never/rarely)				.001		.001
Daily for 30 min			22.69 (2.12, 242.6)		24.06 (2.22, 261.00)	
Visit social place (ref: never/rarely)				.002		.002
Outdoor activity/sport/local facility			0.26 (0.11, 0.63)		0.25 (0.10, 0.62)	
AIC	604.3503		543.5616		538.4052	

Model 1. Household conditions adjusted for common confounders

Model 2. Individual characteristics adjusted for common confounders

Model 3. Household conditions and individual characteristics adjusted for common confounders

*RPR* Relative probability ratio, *LR-p* Likelihood ratio test p-value

After refinement of the household condition model by removing variables no longer showing a significant LR *p*-value of < 0.05 in at least one of the comparisons, bedroom volume, ceiling material, and time since diagnosis of previous TB patient retained overall statistical significance, while household monthly income and family religion were retained irrespective of their statistical significance. A bedroom volume of less than 12m^3^ was associated with case status compared with controls 2 (RPR 3.03 [1.25, 7.33], *p* = 0.012). Ceiling material made from gypsum or plaster rather than wood was associated with case status in comparison with control 1 (RPR 13.62 [3.43, 54.18], LR *p* < 0.001) and control 2 (RPR 3.56 [1.35, 9.40], LR *p* = 0.032). Time since diagnosis of the previous TB patient > 1 year was also strongly associated with case status in both comparisons (RPR 15.39 [7.44, 31.84], *p* < 0.001) and 8.62 [4.39, 16.92], *p* < 0.001) respectively (Model 1).

In the individual characteristics model, having an outdoor occupation (RPR 7.51 [1.43, 38.45], *p* = 0.012 and 13.08 [1.99, 85.33], *p* = 0.003), being underweight compared with normal weight (RPR 4.69 [1.83, 12.04], LR *p* = 0.012 and 5.65 [2.14, 14.88], LR *p* < 0.001), being a sibling parent/grandparent or other relative of the previous patient rather than a spouse when compared with CN1 (RPR 9.70 [2.67, 35.19]; 5.66 [1.64, 19.56]; 4.33 [1.20, 15.62], respectively, LR *p* = 0.002) and being a sibling rather than a spouse when compared with CN2 (5.34 [1.47, 19.39], LR *p* = 0.049), and daily visiting a coffee shop (RPR 10.41 [1.44, 75.48], *p* = 0.008 and 22.69 [2.12, 242.6], *p* = 0.002) were associated with case status in both comparisons. However, visiting other social places such local facilities or playing outdoor sport was inversely associated with case status in comparison with control 2 (RPR 0.26 [0.11, 0.63], *p* = 0.002). In this model also, longer time since diagnosis of the previous TB patient > 1 year was strongly positively associated with case status in both comparisons (RPR 19.38 [8.17, 45.99], *p* < 0.001 and 12.94 [5.66, 29.60], *p* < 0.001, respectively) (Model 2).

Finally, combining the household and individual level models (Model 3) resulted in only slight changes in the magnitudes of association compared with Models 1 and 2. Model 3 showed the best fit as judged by having the lowest AIC value. Based on the constrained likelihood ratio tests, having ceilings of gypsum or plaster rather than wood in comparison with controls 1 (11.24 [2.52, 50.10], LR *p* = 0.002) and small bedroom volume in comparison with controls 2 (3.74 [1.25, 11.20], *p* = 0.016) remained significantly associated with case status. Elapsed time of greater than one year was strongly associated with case status in both comparisons (21.64 [8.73, 53.64], *p* < 0.001 and 13.91 [5.82, 33.24], *p* < 0.001, respectively). Among individual characteristics, having an outdoor occupation, being underweight compared with normal, and frequently visiting a coffee shop were associated with case status in both comparisons. Being overweight compared to normal weight was inversely associated with cases in both comparisons. The previous patient being a sibling, parent/grandparent or other biological relative rather than a spouse was associated with case status in comparison with controls 1, and being a sibling rather than a spouse in comparison with controls 2. Visiting other social places or playing outdoor sports was inversely associated with case status compared with controls 2. Based on constrained models and the likelihood ratio test, none of these variables differed significantly when comparing CN1 with CN2.

To further explore the combined effects of time elapsed and household condition, a binary logistic model including an interaction term between time elapsed and small bedroom volume was constructed in which the two controls were combined into a single group (yielding TB group vs non-TB group) and the model adjusted for ceiling material and the two potential confounding variables (Table 5). The combination was justified on the basis that neither time elapsed nor bedroom volume differed significantly between CN1 and CN2 (0.56 [0.27, 1.15], *p* = 0.113 and 1.52 [0.65, 3.56], *p* = 0.341 respectively), and the combination was performed simply to maximize statistical power. The result is therefore considered only exploratory. The association with small bedroom was evident only among those households with an elapsed time of more than one year (interaction *p* = 0.0014).

**Table 5:** Association of bedroom volume and case status in households with elapsed time of ≤ 1 and > 1 year, combining the two controls and using binary logisticregression adjusted with other variables (ceiling material and common confounders)

Elapsed tie	Bedroom volume (m^3^)	Odds ratio	95% CI	LR- p
≤ 1 year	≥ 12	1	-	
	< 12	0.68	(0.19, 2.48)	.562
> 1 year	≥ 12	1	-	
	< 12	10.76	(2.35, 49.21)	.002

Interaction *p*-value .0014. *LR*-*p*: Likelihood ratio test *p*-value

## Discussion

This study revealed associations between housing characteristics and case/control status. Specifically, small bedroom volume and gypsum/plaster ceiling material were more commonly found among cases than among asymptomatic controls and symptomatic TB-negative controls respectively. These associations persisted after adjustment for individual vulnerability characteristics of the participants and controlling for potential confounding effects of household socioeconomic status and family religion.

However, the design of the study does not explicitly identify the point in the development of active TB at which the condition of the household with a previous TB patient influences the risk of the development of active TB among another household member. That is, whether the household condition influences the potential for initial transmission of the MTB or exerts an effect more indirectly through an increased vulnerability to reactivation of sequestered MTB or increased virulence of the bacteria already present in other household members, could not be established with any certainty.

The effect of small bedroom volume can readily be explained by the increased chances of transmission, and hence initial infection; and similar findings have been reported elsewhere. Thus, a community-based study in Ethiopia reported a similar finding that reduced living space, specifically a living space of less area than 4m^2^ per person, increased the risk of developing TB three-fold [28].

It is noted that the positive association of having a small bedroom with case status was stronger and significant only when compared with controls 2 and not with controls 1. This suggests that controls 1, that is, patients with respiratory symptoms similar to, but not actually, those of TB, may share this risk factor with TB patients. Furthermore, having a small bedroom did not differ significantly between the two control groups in the multivariable models.

Why the living room ceiling material of gypsum or plaster should be positively associated with case status compared with control 1 is less obvious. However, it has been reported that building materials can affect dust accumulation [29, 30], microbial growth and mould [31]. Lower-cost materials such as plasterboard or gypsum may foster dampness and microbial persistence, and experimental work has shown that while wood and lime plaster resist fungal growth, tile and plaster mortar allow it [32]. The release of fungal spores may contribute to the dust load of room air [33, 34]. Dust can also be released directly from certain building materials. Sawing and sanding of gypsum and plaster board during construction can release dust, including small amounts of crystalline silica particles [35], but the generation of dust from such boards once fitted to ceilings seems unlikely.

Regardless of its composition, airborne dust is reported to facilitate TB transmission. Mycobacterium smegmatis, a surrogate marker of MTB, was shown to survive and remain viable after 19 days when attached to dust [36]. Furthermore, breathing fine dust, especially that containing silica particles, can have harmful effect of the respiratory tract, weakening the immune response and thereby increasing vulnerability to TB infection. Exposure to PM_2.5_ has been linked to the development of active TB from LTBI in China [37].

A notable finding was that the time elapsed since the presence of a TB patient in the household of more than one year was more common among cases than among either of the controls. This may reflect the situation in which active TB, either primary TB or reactivation of LTBI, can develop at a variable length of time after initial infection. This result is consistent with evidence from a study in Peru [38], in which about half of the newly diagnosed TB patients reported having a previous household TB case within the past 1–10 years. This suggests that following an earlier exposure to a TB patient in the household there was sufficient time for the disease to develop before diagnosis. In the present study, 23% of cases reported an elapsed time of more than 10 years compared with only 2% and 5% of CN1 and CN2 (data not shown), indicating that even long-past exposure may contribute to reactivation of latent infection.

Closeness of contact can favour transmission. A study in Thailand found that the type of relationship influenced TB risk. Spouses and parents, who spent more prolonged and intimate time with patients, had higher odds of LTBI compared to other household members [39], which contrasts slightly with our finding of case status being positively associated with sibling, parent/grandparent and other biological relative rather than spouse. The strongest association was for the sibling relationship. This may reflect a cultural difference in which Indonesian siblings commonly sleep the same room due to limited housing space [40], which could increase opportunities for close contact.

Regarding individual vulnerability factors identified in the current study, frequent visits to a coffee shop and closeness of contact with the previous patient are not unexpected. While males were more common among cases, the effect was eclipsed in the multivariable environment after inclusion of having an outdoor occupation and frequency of coffee shop visits. In the local context, males are more likely to go outside for work and to frequent rural traditional coffee shops (*warung kopi)* [41]. These local coffee shops are mostly small, indoor establishments, frequently crowded with local male residents. Visiting such a venue may increase the probability of being infected from an external source, not related to the previous TB patient in the home [24].

On the other hand, the findings show an inverse association between case status and having outdoor activities such as playing sport. This may reflect reduced participation as a consequence of their health condition − in other words, a potential instance of reverse causality. A similar explanation may underlie the association of underweight with case status, as also reported in previous studies from Ethiopia [12] and China [42].

### Strengths and limitations

This study adds evidence that both household and individual factors can be associated with the development of active TB among members of households having experienced a prior TB patient in the home. Using two control groups helped to distinguish household conditions linked to the development of symptomatic TB itself from those linked with respiratory symptoms only suggestive of, but not actually resulting from, TB. Combining household observation and measurements with direct interviews helped to validate the data collected.

However, several limitations must be noted. First, this study did not specify the age of the previous TB patient in the household, apart from recording the relationship with the study participant. This may have introduced some bias as the transmission dynamics of childhood and adult TB are known to differ [43–45]. A related issue is the failure to record explicitly whether the participant was a contact, that is living in the household at the same time as the prior TB patient. However, a generated variable identifying participants as probably living in the house at the same time as the previous patient vs definitely or possibly not living in the house at the same time failed to indicate a significant association with case/control status in the multivariable setting after adjusting for time elapsed. Second, asymptomatic household controls (CN2) were not tested with chest radiography or microbiological testing (e.g., Xpert MTB/RIF), or with tuberculin skin test (TST) or IGRA, thus undiagnosed subclinical tuberculosis or LTBI, which are prevalent in high-burden settings, could not be ruled out.

Third, it was not possible to know whether the newly diagnosed cases represented reactivation of LTBI or were new primary TB cases. Future studies may include additional testing, such as the TST or IGRA, to detect existing LTBI in participants. This would help to confirm the non-respiratory-symptom participants as infection-free and also to distinguish between reactivation of latent infection and new recent infection among cases.

Fourth, the number of previous TB patients in each household was not recorded, and it might be expected that more than one prior TB patient would further increase the risk of a subsequent case. The data collected related only to the latest prior TB patient in the home.

Fifth, environmental measurements of temperature, relative humidity, carbon dioxide levels, and air speed in the household could only be considered as true at the time of measurement. Time and resource limitations made it unfeasible to conduct measurements over multiple days as originally intended. Lastly, the sample size was small, leading to poor precision of some estimated associations and low power to detect weak associations.

Additionally, BMI and social activity were measured after enrolment and may be a consequence of the current health condition, even though the question pertaining to social activity was posed in the framework of the participant’s habitual activity.

## Conclusion

Household conditions and individual vulnerability characteristics were associated with TB risk. Ceilings made of gypsum or plaster, and having a biological non-spousal relationship with the previous case distinguished TB-positive from symptomatic TB-negative participants. Small bedroom size distinguished TB-positive cases from asymptomatic participants. Elapsed time of more than one year since diagnosis of the previous household TB patient, frequent visits to local coffee shops and outdoor occupation distinguished TB-positive cases from both symptomatic TB-negative and asymptomatic participants.

Although housing conditions are not easily modified, they may be used as indicators to identify households at potentially higher risk of TB among members where there is a history of previous household TB. Ceiling material and bedroom size could be considered as practical markers for prioritizing screening and follow-up. These findings suggest some interventions, such as improving bedroom space, structural improvements and targeted screening in households with prior TB, especially after one year. Collaboration between the National TB Programme and the Housing Department could support the development of healthy housing guidelines that reflect the household conditions commonly seen among these TB patients.

## Supplementary Information

Supplementary Material 1.

## Data Availability

The original contributions presented in the study are included in the article. Further inquiries can be directed to the corresponding author. The questionnaire was developed specifically for this study.

## Abbreviations

AFB: Acid-Fast Bacillus (smear test)
AIC: Akaike Information Criterion
BMI: Body Mass Index
CN1: Control 1 (Symptomatic non-TB control)
CN2: Control 2 (Non-respiratory symptom control)
CO₂: Carbon Dioxide
CS: Case (Symptomatic TB-positive Participant)
DR: Drug-resistant
DS: Drug-sensitive
EC Number: Ethical Clearance Number
IGRA: Interferon Gamma Release Assay
LR: Likelihood Ratio
LR p: Likelihood Ratio p-value
LTBI: Latent TB Infection
MTB: Mycobacterium tuberculosis
PM_2.5_: Particulate Matter ≤ 2.5 µm
Puskesmas: Primary Health Care Centre (Indonesia)
RIF: Rifampicin
RPR: Relative Probability Ratio
TB: Tuberculosis
TST: Tuberculin Skin Test
Xpert MTB/RIF: Xpert Mycobacterium tuberculosis/Rifampicin
