# Supplementary material for: The association of household conditions with the development of active pulmonary TB among in households with a previous TB patient: a community-based case–control study
Source: BMC Pulm Med. 2026 Jun 26;26:410. doi: 10.1186/s12890-026-04434-5 (PMC13573435; doi:10.1186/s12890-026-04434-5)
Supplement: Supplementary file 1 [file 12890_2026_4434_MOESM1_ESM.docx]

**Supplementary material**

**“The association of household conditions with the development of active TB among household contacts: A community-based case-control study”**

Date……………………	**Unique ID** [	] [	] [	] [	]

| A. **SCREENING QUESTIONNAIRE** | | |
| --- | --- | --- |
| This question is a screening question to ensure the participant has ever lived in the same household as a TB patient.  *Please explain our study briefly (Study aim, procedure).* | | |
| **No** | **Questions** | **Answers (Please tick)** |
| G1 | Do you ever had household contact with a TB patient? | [ ] 1. Yes  [ ] 0. No, exclude from the participant  [ ] 88.Not sure, exclude from study |
| G2 | If yes, how long have you shared a bedroom with that TB patient? | _____ days /month/year |
| G3 | What was the relationship of that patient to you? | [ ] 1. Grandfather/grandmother  [ ] 2. Spouse  [ ] 3. Sibling  [ ] 88. Other ______ |
| G4 | For how long were they treated? | ____ months |
| G5 | What was/is the type of TB | [ ] 1. Multi-drug resistant (MDR) TB  [ ] 0. Drug-susceptible TB  [ ] 88. Not sure |
| G6 | What was the result of treatment of the TB patient? | [ ] 1. Cured  [ ] 2. Completed treatment  [ ] 3. Relapsed (TB again later)  [ ] 4. Died  [ ] 5. Defaulted (did not complete the treatment)  [ ] 88.Not sure |

| B. **MAIN QUESTIONNAIRE** | | | |
| --- | --- | --- | --- |
| I. **INDIVIDUAL CHARACTERISTICS** | | | |
| **Section A. Demographic characteristics.** | | | |
| A1a | Unique ID | | [ ] [ ] [ ] [ ] |
| A1b | Type of participant | | [ ] 1. Case  [ ] 2. Control 1 (with symptoms)  [ ] 3. Control 2 (without symptoms) |
| A2 | Sex | | [ ] 1. Male  [ ] 2. Female  [ ] 99. Refuse to answer |
| A3 | Age in years (If don’t know, write 888, if refuse to answer, write 999) | | [ ] [ ] [ ] |
| A4 | Occupation | | [ ] 1. Civil servant/state enterprise  [ ] 2. Student  [ ] 3. Small-scale vendor / service provider  [ ] 4. Business owner / entrepreneur  [ ] 5. Laborer / manual worker  [ ] 6. Retired / homemaker / housewife  [ ] 7. Agriculture / fishery  [ ] 8. Unemployed  [ ] 99. Refuse to answer |
| A5 | Religion | | [ ] 1. Islam  [ ] 2. Christianity  [ ] 77. Other  [ ] 99. Refuse to answer |
| A6 | Marital status | | [ ] 1. Single  [ ] 2. Married with children  [ ] 3. Married, no children  [ ] 4. Widowed / divorced / separated  [ ] 99. Refuse to answer |
| A7 | Highest level of education completed | | [ ] 1. Never went to school  [ ] 2. Primary school  [ ] 3. Junior high school  [ ] 4. High school  [ ] 5. Bachelor's degree  [ ] 6. Higher than bachelor's degree  [ ] 88. Not sure  [ ] 99. Refuse to answer |
| A8 | Residential (subdistricts) | | [ ] 1. Koto Tangah  [ ] 2. Bungus Teluk Kabung  [ ] 3. Kuranji  [ ] 4. Lubuk Begalung  [ ] 5. Lubuk Kilangan  [ ] 6. Nanggalo  [ ] 7. Padang Barat  [ ] 8. Padang Selatan  [ ] 9. Padang Timur  [ ] 10. Padang Utara  [ ] 11. Pauh |
| A9 | Referral Hospital / Public Health Centre | | [ ] 1. Bungus  [ ] 2. Lubuk Kilangan  [ ] 3. Lubuk Begalung  [ ] 4. Pegambiran  [ ] 5. Seberang Padang  [ ] 6. Pemancungan  [ ] 7. Rawang  [ ] 8. Andalas  [ ] 9. Padang Pasir  [ ] 10. Ulak Karang  [ ] 11. Air Tawar  [ ] 12. Alai  [ ] 13. Nanggalo  [ ] 14. Lapai  [ ] 15. Belimbing  [ ] 16. Kuranji  [ ] 17. Ambacang  [ ] 18. Pauh  [ ] 19. Air Dingin  [ ] 20. Lubuk Buaya  [ ] 21. Ikur Koto  [ ] 22. Anak air  [ ] 23. Hospital, please specify _____________­­­­­_____________ |
| A10 | Geo Location | | __ |
| A11 | Phone Number | | [ ][ ][ ][ ][ ][ ][ ][ ][ ][ ][ ][ ] |
| **Section B. Clinical assessment** | | | |
| **B1. TB diagnosis** | | |  |
| B1a | | Chest x-ray result | [ ] 0. Negative  [ ] 1. Positive |
| B1b | | Sputum GeneXpert MTB/RIF  Result | [ ] 0. Negative  [ ] 1. Positive |
| B1c | | AFB Smear Microscopy Result | [ ] 0. Negative  [ ] 1. Positive |
| **B2. Physical Examination.** | | |  |
| B2a. Nutritional Status | | |  |
| B2a1 | Weight | | [ ][ ][ ] kg |
| B2a2 | Height | | [ ][ ][ ] cm |
| B2b. Underlying disease | | |  |
| B2b | Do you have any medical conditions? (Multiple answers allowed; Read each one to the participant, and wait for a yes/no response. If possible check-in medical records) | | [ ] 0. None or no Disease  [ ] 1. Diabetes  [ ] 2. Hypertension  [ ] 3. Hyperlipidemia  [ ] 4. Tuberculosis  [ ] 5. Chronic kidney disease  [ ] 6. Asthma  [ ] 7. COPD  [ ] 8. Other respiratory disease  [ ] 9. Other chronic disease  [ ] 99. Refuse to answer |
| **Section C. Individual Vulnerability Status** | | |  |
| C1 | History of BCG vaccine | | [ ] 0. No  [ ] 1. Yes  [ ] 88.Not sure |
| C2 | HIV status | | [ ] 0. No  [ ] 1. Yes  [ ] 88. Not sure |
| C3 | Diabetes Mellitus status | | [ ] 0. No  [ ] 1. Yes  [ ] 88. Not sure |
| C4 | History of TB | | [ ] 0. No  [ ] 1. Yes  [ ] 88. Not sure |
| C5 | History of incomplete treatment for TB | | [ ] 0. No  [ ] 1. Yes  [ ] 88. Not sure |
| C6 | Have you ever been diagnosed with latent TB infection (LTBI) by doctor or have positive test for TST (Tuberculin Skin Test) | | [ ] 0. No  [ ] 1. Yes  [ ] 88.Not sure |
| **Section D. External Exposure** | | |  |
| **D1. Social activity** | | |  |
| D11 | What was the usual frequency of your visits to a coffee shop or bar on a daily basis of at least 30 minutes? | | [ ] 1. Daily in 30 minutes  [ ] 2. Go to a coffee shop or drink coffee weekly  [ ] 3. Less than weekly  [ ] 4. Never |
| D12 | How many people were generally in the café when you visited?  (If not sure or not sure, write 88; if you refuse to answer, write 99). | | [ ][ ][ ] |
| D13 | Were the people in the café generally close to each other (≤1 meter)? | | [ ] 1. Yes  [ ] 0. No  [ ] 88.Not sure |
| D14 | What was the main place for other outside habitual activity? | | [ ] 1. Mosque / Cultural Hall  [ ] 2. Corner shop  [ ] 3. Nearby vegetable market  [ ] 4. Neighbour’s home  [ ] 5. Outdoor activity/sport  [ ] 6. Seldom go out. |
| **D2. Mobility** | | |  |
| D21 | The frequency of trips from home residence. | | [ ] 0. No  [ ] 1. Yes  [ ] 88. Not sure |
| D22 | Mobility of travelling ≥2 times per month. | | [ ] 0. No  [ ] 1. Yes  [ ] 88. Not sure |
| D23 | Commonly used mode of transport when travelling | | [ ] 0. No  [ ] 1. Yes  [ ] 88. Not sure |
| D24 | How many people are in the transportation? Enter 88 if not sure. | | [ ][ ][ ] people |
| D25 | Do you spend ≥3 hours away when travelling? | | [ ] 0. No  [ ] 1. Yes  [ ] 88. Not sure |
| D26 | Have you ever spent nights away from your primary residence in the past six months? | | [ ] 1. Yes  [ ] 0. No  [ ] 88. Not sure |
| D27 | If yes, how many | | [ ] [ ] nights |

| **II. FAMILY CHARACTERISTICS** | | |
| --- | --- | --- |
|  | ***Family economic status*** |  |
| E1 | Household monthly income (if not sure, write 88; if refuse to answer, write 99). | [ ] 1. <Rp. 500.000  [ ] 2. Rp.500.000-2.500.000  [ ] 3. Rp.2.500.000-5.000.000  [ ] 4. Rp.>5.000.000  [ ] 99. Refuse to answer |
| E2 | How many members are there in your household including yourself? (sleep under the same roof; if refuse to answer, write 99) | [ ][ ] Persons |
| E3 | Do you have Air Conditioning in the bedroom. | [ ] 0. No  [ ] 1. Yes  [ ] 88. Not sure |
| E4 | If yes, do you use AC most days? | [ ] 0. No  [ ] 1. Yes  [ ] 88. Not sure |
|  | ***Smoking inside the house*** |  |
| E5 | Do you have a history of smoking? | [ ] 0. No  [ ] 1. Yes  [ ] 88. Not sure |
| E6 | Do you currently smoke? | [ ] 1. Yes (≥ 1 cigarette in the past month)  [ ] 2. No (have not smoked in the past 6 months)  [ ] 3. Just quit (less than 6 months ago. |
| E7 | How often do you smoke? | [ ] 1. Daily  [ ] 2. Weekly  [ ] 3. Monthly |
| E8 | Are there other people who smoke inside the house? | [ ] 1. No  [ ] 2. Yes, but smoke less than daily  [ ] 3. Yes, and smoke daily. |
| **III. HOUSING CONDITION** | |  |
| **F1.** **House Characteristics** | | Instruction: Measure or estimate the dimensions of the house, living room, and bedroom. Walk inside the household and make estimates of overcrowding, ventilation, thermal comfort, CO_2_ levels, and dampness. Obtain data by direct measurements at home visit. |
|  | ***Whole house*** |  |
| F11a | What are the length, width and height of the house?  (length, width, height) | [ ][ ].[ ] x [ ][ ].[ ] x [ ][ ].[ ] m |
| F12a | Type of housing | [ ] 1. Traditional wooden house (Minangkabau)  [ ] 2. Modern House  [ ] 3. Dormitory |
| F12b | Number of storeys | [ ] 1. One story  [ ] 2. Two stories  [ ] 3. ≧3 stories |
| F12c1 | Is the ground storey raised above the ground? | [ ] 0. No  [ ] 1. Yes  [ ] 88. Not sure |
| F12c2 | If yes, how high is the floor of the house from the ground? | [ ].[ ] metres |
| F12d | If yes, what is inside of that space? | ___________ |
| F12e | Roof material | [ ] 1. Tile  [ ] 2. Metal  [ ] 3. Wood  [ ] 4. Asphalt shingles  [ ] 5. Other (specify) _____________ |
| F12f | Ceiling material | [ ] 1. Gypsum  [ ] 2. Wood  [ ] 3. Plaster  [ ] 4. Other (specify) _____________ |
| F12g | Floor Material | [ ] 1. Sand  [ ] 2. Wood  [ ] 3. Ceramic  [ ] 4. Other (specify) _____________ |
| F12h | Wall material | [ ] 1. Tile  [ ] 2. Wood  [ ] 3. Ceramic  [ ] 4. Other (specify) ______________ |
| F12i | House form | [ ] 1. Attached  [ ] 2. Apartment  [ ] 3. Detached |
|  | ***Living room*** |  |
| F11b | What are the length, width and height of the living room? (length, width, height) | [ ][ ].[ ] x [ ][ ].[ ] x [ ][ ].[ ] m |
| F11c | How long do you (the participant) stay in the living room each day? | [ ][ ].[ ] hours |
|  | ***Bedroom*** | Ask the participant to estimates their bedroom’s size by themselves. |
| F13a | What are the length and width of your bedroom? length and width) | [ ][ ][ ] x [ ][ ][ ] m |
| F13b | How many people normally sleep in your bedroom? | [ ] 1. <3 persons  [ ] 2. 3-4 persons  [ ] 3. 5-6 persons  [ ] 4. ≥7 persons |
|  | ***Pet in the house*** |  |
| F14a | Do you have a pet animal? | [ ] 0. No  [ ] 1. Yes  [ ] 88. Not sure |
| F14b | The type of pet. | [ ] 1. Chicken  [ ] 2. Cat  [ ] 3. Dog  [ ] 4. Cow  [ ] 5. Goat  [ ] 6. Other_________________________________ |
| F14c | Does/do the pet/pets go inside the bedroom? | [ ] 0. No  [ ] 1. Yes, type of pet _______  [ ] 88. Not sure |
|  | ***Ventilation habit*** |  |
|  |  | Count or enquire about the number of windows and ventilation habits in the living room and bedroom. Please prepare the instrument |
| F15a | The number of windows in the living room | [ ][ ] |
| F15b | Practice of opening the windows in the living room (in the past 12 months). | [ ] 1. No window  [ ] 2. Never open  [ ] 3. Seldom open (less than 3 days/week)  [ ] 4. Open every day |
| F15c | The number of windows in the bedroom | [ ][ ] |
| F15d | Practice of opening the windows in the bedroom (in the past 12 months) | [ ] 1. No window  [ ] 2. Never open  [ ] 3. Seldom open (less than 3 days/week)  [ ] 4. Open every day |
| **F2. Overcrowding** | |  |
| F2 | Overcrowding | Instructions: please prepare the tools |
| F21 | CO_2_ level at time of home visit. | [ ][ ][ ] ppm |
| **F3. Thermal Comfort** | |  |
| F31 | Time |  |
| F32 | Participant’s clothing (based on the picture) | [ ][ ][ ] |
|  |  | |
| F33 | Participant’s activity level (at or just before the tie of the home visit) | [ ] 1. Sleeping  [ ] 2. Reading or writing  [ ] 3. Standing in house  [ ] 4. Walking about in house  [ ] 5. Housecleaning  [ ] 6. Cooking  [ ] 7. Socializing  [ ] 8 Other specify __________ |
| F34 | How do you feel about today's weather? | [ ] 1. Hot  [ ] 2. Warm  [ ] 3. Slightly warm  [ ] 4. Neutral  [ ] 5. Slightly cool  [ ] 6. Cool  [ ] 7. Cold |
| F35 | Outside Relative Humidity | [ ][ ].[ ] % |
| F36 | Temperature: | [ ][ ].[ ] ^O^ C |
| F37 | Air speed | [ ][ ].[ ] m/s |
| F38 | Radiant temperature | [ ][ ].[ ] ^O^ C |
| F39a | Total number of occupants in the living room now (current situation) . | [ ][ ] |
| F39b | Total number of occupants in the living room normally. | [ ][ ] |
| F310 | Participant’s location in living room. (Place an “X” in the approximate place where you most often work). Please indicate the direction of air flow within the room using an arrow. |  |
| **F4. Dampness** | |  |
| F4 | Dampness | Instructions: please observe the physical condition of living room |
| F41 | Water leakage: Did you have flooding and/or water leakage during the past year or earlier in living room? | [ ] 0. No  [ ] 1. Yes, a little  [ ] 2. Yes, a lot  [ ] 88. Not sure |
| F42 | Floor moisture: Is there any loosening, bubbling or discoloration of the flooring material in the living room.? | [ ] 0. No  [ ] 1. Yes, big  [ ] 2. Yes, small  [ ] 88. Not sure |
| F43 | Visible dampness: Is there any visible mould or damp spots in the living room? | [ ] 0. No  [ ] 1. Yes, big  [ ] 2. Yes, small  [ ] 88. Not sure |
| **F5. Garbage** | |  |
| F51 | What type of collection/storage place for wet (organic) waste in the house? | [ ] 1. Closed trash can  [ ] 2. Open trash can  [ ] 88.Not sure |
| F52 | What are the main ways of handling the waste? | [ ] 1. Transported by officers, compost it  [ ] 2. Burn it  [ ] 3. Throw it away outside the house  [ ] 4. Other specify ______________________  [ ] 88. Not sure |
| **F6. Handwashing** | |  |
| F61 | Do you wash your hands regularly? | [ ] 0. No  [ ] 1. Yes  [ ] 88. Not sure |
| F62 | Do you always wash your hands using clean water? | [ ] 0. No  [ ] 1. Yes  [ ] 88. Not sure |
| F63 | Is there a place to wash your hands? | [ ] 1. Sink/ tap water/gallon  [ ] 2. Bucket using a dipper  [ ] 88. Not sure  [ ] 99. Other specify |
| F64 | What types of hand washing facilities are available? | [ ] 0. No  [ ] 1. Yes  [ ] 88. Not sure |
| F65 | Is water available at hand washing facilities? | [ ] 0. No  [ ] 1. Yes  [ ] 88. Not sure |
| F66 | Is soap, detergent or antiseptic liquid available at the hand washing place? | [ ] 0. No  [ ] 1. Yes  [ ] 88. Not sure |
| **F7. Cooking fuel** | |  |
| F71 | What type of cooking fuel do you primarily use at home? | [ ] 1. Electricity  [ ] 2. Gas (LPG)  [ ] 3. Kerosene  [ ] 4. Wood  [ ] 5. Charcoal  [ ] 88. Not sure |
